# Sex and Pubertal Differences in the Maturational Trajectories of Sleep Spindles in the Transition from Childhood to Adolescence: A Population-Based Study

**DOI:** 10.1523/ENEURO.0257-21.2021

**Published:** 2021-07-14

**Authors:** Anna Ricci, Fan He, Susan L. Calhoun, Jidong Fang, Alexandros N. Vgontzas, Duanping Liao, Edward O. Bixler, Magdy Younes, Julio Fernandez-Mendoza

**Affiliations:** 1 Sleep Research and Treatment Center, Department of Psychiatry and Behavioral Health, Penn State College of Medicine, Hershey, PA17033; 2 Department of Public Health Sciences, Penn State College of Medicine, Hershey, PA17033; 3 Sleep Disorders Centre, University of Manitoba, Winnipeg, ManitobaR3M 0A7, Canada

**Keywords:** adolescence, brain maturation, childhood, developmental biology, puberty, sleep spindles

## Abstract

Sleep spindles, bursts of electroencephalogram (EEG) activity in the σ-frequency (11–16 Hz) range, may be biomarkers of cortical development. Studies capturing the transition to adolescence are needed to delineate age-related, sex-related, and pubertal-related changes in sleep spindles at the population-level. We analyzed the sleep EEG of 572 subjects 6–21 years (48% female) and 332 subjects 5–12 years (46% female) followed-up at 12–22 years. From 6 to 21 years, spindle density (*p* quadratic = 0.019) and fast (12–16 Hz) spindle percent (*p* quadratic = 0.016) showed inverted U-shaped trajectories, with plateaus after 15 and 19 years, respectively. Spindle frequency increased (*p* linear < 0.001), while spindle power decreased (*p* linear < 0.001) from 6 to 21 years. The trajectories of spindle density, frequency, and fast spindle percent diverged between females and males, in whom density plateaued by 14 years, fast spindle percent by 16 years, and frequency by 18 years, while fast spindle percent and spindle frequency continued to increase until 21 years in females. Males experienced a longitudinal increase in spindle density 31% greater than females by 12–14 years (*p* = 0.006). Females experienced an increase in spindle frequency and fast spindle percent 2% and 41% greater, respectively, than males by 18–22 years (both *p* = 0.004), while males experienced a 14% greater decline in spindle power by 18–22 years (*p* = 0.018). Less mature adolescents (86% male) experienced a longitudinal increase in spindle density 36% greater than mature adolescents by 12–14 years (*p* = 0.002). Overall, males experience greater maturational changes in spindle density in the transition to adolescence, driven by later pubertal development, and sex differences become prominent in early adulthood when females have greater spindle power, frequency, and fast spindle percent.

## Significance Statement

Age-related changes in sleep spindles reflect maturation of the thalamocortical network. We provide evidence that spindle metrics follow distinct developmental trajectories from each other and previously described sleep oscillations shown to index brain maturation in the transition to adolescence. Importantly, we report novel data regarding the association between spindle activity and pubertal development. Specifically, we found that less mature adolescents (86% male) experienced a greater increase in spindle density in the transition to ages 12–14 years, while more mature adolescents (75% female) experienced a greater decline in σ/spindle power by the same age. These data suggest that females, who enter pubertal development earlier than males, may also begin brain maturational processes in the thalamocortical network at an earlier stage than males.

## Introduction

Sleep spindles occur as synchronized bursts of sinusoidal waves in the σ-frequency range (11–16 Hz) and are electroencephalogram (EEG) hallmarks of stage 2 (N2) of non-rapid eye movement (NREM) sleep (Rechtschaffen and Kales, 1968; Iber et al., 2007). Spindles are generated in the thalamus and synchronized in the cortex and thus, reflect activity of the thalamocortical network (Steriade et al., 1985; Steriade,1999). A predominant role of spindles is protecting the sleeping brain from external sensory stimuli, serving as biomarkers of sleep integrity (Dang-Vu et al., 2010, 2015; Saletin et al., 2013). Moreover, age-related changes in spindle activity may signal maturation of the thalamocortical network (D’Atri et al., 2018; Guadagni et al., 2021). It has also been suggested that spindles support cognitive function by preventing sleep fragmentation, allowing for better off-line information processing (e.g., memory consolidation), which is critical during early development (Fogel and Smith, 2011; Gruber and Wise, 2016). Indeed, spindles have been associated with cognitive processes in children and adolescents (Geiger et al., 2011; Chatburn et al., 2013; Hoedlmoser et al., 2014); however, such relationship differs across the lifespan (Reynolds et al., 2018). As sleep spindles are purported EEG biomarkers of neurodevelopment that represent the strength/integrity of the thalamocortical network, it is essential to examine their maturational trajectories and potential sex differences across specific developmental stages. These data will inform and expand the knowledge of sleep spindles as they relate to cognition and neurodevelopment in youth.

The majority of previous studies on the maturational trajectories of sleep spindles have relied on σ power as a surrogate marker of spindle activity (Gaudreau et al., 2001; Campbell et al., 2005; Tarokh and Carskadon, 2010; Tarokh et al., 2011; Baker et al., 2012, 2016; Prehn-Kristensen et al., 2013; Olbrich et al., 2017). Campbell and Feinberg (2016) found a longitudinal increase in σ power from 6 to 12 years, followed by a decline from 12 to 16 years (*n* = 92). While these σ power trajectories may generalize to spindles, studies examining age-related changes specifically for spindles suggest that different metrics undergo distinct developmental trajectories (Bòdizs et al., 2014; Nader and Smith, 2015; McClain et al., 2016). Hahn et al. (2019) observed a longitudinal increase in spindle density and frequency that appeared to be driven by fast (13–15 Hz) spindles in the transition from 8–11 to 14–18 years (*n* = 34). Although Hahn et al. (2019) did not examine sex or pubertal differences, Goldstone et al. (2019) found that females had higher fast (∼13 Hz) spindle frequencies than males, and more mature adolescents had greater fast spindle density than less mature adolescents in their cross-sectional sample (*n* = 134, 12–21 years). A more recent study found higher spindle amplitude, frequency, and density in 31 girls compared with 30 boys (9–14 years), suggesting greater thalamocortical coherence in females than males (Markovic et al., 2020). Finally, an extensive analysis of spindle activity (11–15 Hz) in 11 630 individuals (Purcell et al., 2017) found distinct life-course trajectories and sex differences in spindle metrics from ages 4–97, including 448 children (4–10 years) with obstructive sleep apnea followed-up after six months, a cross-sectional sample of 509 adolescents (16–19 years), and a cross-sectional family sample with 183 youth (7–23 years). Purcell et al. (2017) produced seminal data from a life-course perspective; however, studies are still needed to expand on their findings by examining cohorts with greater representation of early adolescents undergoing puberty and longer longitudinal follow-up periods capturing the developmental transition to adolescence.

The aim of the present study was to improve the knowledge of age, sex, and pubertal differences in sleep spindles and related σ activity during this developmental period by examining sex differences in 572 subjects aged 6–21 years, and sex-related and pubertal-related differences in 332 subjects aged 5–12 years followed-up 6–13 years later at ages 12–22 years. As different spindle characteristics may represent specific features of cortical development, we examined spindle density, frequency, and power, as well as the percent of fast spindles. Spindle density may reflect thalamocortical coherence/connectivity (Steriade et al., 1985), while spindle frequency polarization of thalamocortical neurons (Goldstone et al., 2019), spindle power white matter integrity around the thalamus (Fernandez and Lüthi, 2020), and fast spindles hippocampal development (Saletin et al., 2013). Thus, examining each of these metrics is essential to understand neurodevelopmental changes in sleep spindles. We compared the trajectories of spindle metrics to σ power as it has been used as a surrogate measure of spindle activity. While we expect females to have greater spindle activity than males as indicated by previous studies (Purcell et al., 2017), we also hypothesize that age-related changes in spindle activity will be associated with pubertal development.

## Materials and Methods

### Penn State child cohort (PSCC)

The PSCC is a randomly-recruited sample of 700 children (47.7% female, 23.7% racial/ethnic minority) from the general population who underwent a comprehensive in-lab study between ages 5 and 12 (median 9 years; Bixler et al., 2008; Calhoun et al., 2014). Out of the 700 children, 421 returned 6–13 years later (median 7.4 years) for a follow-up study when they were 12–23 years (median 16 years, 46.1% female, 21.9% racial/ethnic minority; Bixler et al., 2016; Fernandez-Mendoza et al., 2016, 2017). A total of 279 subjects (median 9 years, 47.8% female, 29.3% racial/ethnic minority) did not return for the follow-up study but were not significantly different from the 421 who were followed-up in their demographic characteristics at ages 5–12 (Bixler et al., 2016). All subjects or parents/legal guardians provided informed written consent for the study protocol, which was approved by Penn State’s Institutional Review Board.

### Demographics and clinical measures

During baseline and follow-up visits, the sleep study consisted of a clinical history, physical examination, self-reported questionnaires and a one-night, 9-h polysomnography (PSG) recording, all of which occurred in sound-controlled, light-controlled, and temperature-controlled rooms. Sex, race, ethnicity, and date of birth were reported during the clinical history at baseline. Height and weight were measured during the physical examination and body mass index (BMI) percentile was calculated (Kuczmarski et al., 2002). A 3-h standardized neurobehavioral assessment was performed at both time points (Achenbach, 1991; Wechsler, 1991, 1997, 1999, 2003; Achenbach and Rescorla, 2003). During the clinical history, parents at baseline and/or subjects at follow-up reported on the presence of a lifetime history of any psychiatric or behavioral disorder (Frye et al., 2018). Medication use was reported by parents at baseline and/or by subjects at follow-up during the clinical history and on an evening questionnaire (Frye et al., 2018). Psychotropic medication use was defined by a report of stimulants, antidepressants, anxiolytics, antipsychotics and/or hypnotics (*n* = 58 at baseline and *n* = 67 at follow-up). At follow-up, Tanner stage was ascertained via a self-rating scale (Carskadon and Acebo, 1993) providing stages 1 (prepuberty), 2 (early puberty), 3 (mid puberty), 4 (late puberty), and 5 (adulthood).

### PSG

Registered PSG technicians (RPSGTs) applied electrodes to the subject’s scalp, face, and legs at 9 P.M. All sleep data were recorded for 9 h of time in bed from the time of “lights out” (9–11 P.M.) until the time of “lights on” (6–8 A.M.) to accommodate to the subject’s habitual sleep schedule. All sleep data at baseline and follow-up were recorded using Grass PSG equipment (Grass-Telefactor) and included EEG, electrooculography, electromyography, electrocardiography (ECG) and respiratory measures. All PSG recordings were visually scored by RPSGTs in 30-s epochs following standard criteria (Rechtschaffen and Kales, 1968; Iber et al., 2007; Bixler et al., 2008, 2016). Out of the 700 recordings at baseline (B), 48 were performed on paper PSG and six digital records were corrupted, thus, their EEG could not be analyzed. All 421 recordings at follow-up (F) were performed on digital PSG and were analyzable.

There were unavoidable PSG system updates during this large, long-term study that collected baseline data across four years and follow-up data 6–13 years later across three years. Given these PSG updates there were differences in the number of EEG channels, adding other referencing methods, filter settings, and sampling rates, all of which were accounted for in the spectral analyses as well as in the statistical analyses by controlling for the PSG system (see below, Statistical analyses). Specifically, a total of 373 baseline files (B1) were recorded with a sampling rate of 100 Hz and filter settings at 0.1–100.0 Hz. The remaining 273 baseline files (B2) were recorded with sampling rates at 100.0 and 200.0 Hz and filter settings at 0.01–30.0 Hz. Importantly, there were no significant differences at baseline between subjects recorded with PSG system B1 versus B2 in terms of critical demographic factors, including distribution of male sex (49.6% vs 52.3%, *p* = 0.700) and age (8.4 ± 1.7 vs 8.5 ± 1.7, *p* = 0.799). All of the follow-up PSG files (F) were recorded with a sampling rate of 200 Hz and filter settings at 0.1–70.0 Hz. All PSG data were automatically processed with spectral analysis software with the common EEG frequencies ranging between 0.3 and 30.0 Hz, thus, activities below and above this range were removed for consistent data processing across all European data format (EDF) records from different PSG systems. Additionally, not all subjects had available central derivations with contralateral referencing and spectral analyses for those subjects were conducted using the central derivations with ipsilateral referencing used at the time of the PSG (only 122 in cross-sectional analyses and 167 in longitudinal analyses in the present study). Among the subset of records that had both contralateral and ipsilateral channels, we found excellent concordance in both the cross-sectional sample [C3-M1 vs C3-M2, *n* = 105, ρ_c_ = 0.985, 95% confidence interval (95%CI) = 0.979, 0.990; C4-M1 vs C4-M2, *n* = 109, ρ_c_ = 0.983, 95%CI = 0.975, 0.988] and longitudinal sample (C3-M1 vs C3-M2, *n* = 188, ρ_c_ = 0.997, 95%CI = 0.996–0.998; C4-M1 vs C4-M2, *n* = 190, ρ_c_ = 0.996, 95%CI = 0.995, 0.997) assuring reliability of the data.

All 646 baseline and 421 follow-up (*N* = 1067) digital PSG records were converted into EDF files and analyzed in a blind manner using two independent systems: Michele Sleep Scoring (MSS; Cerebra Health, Sleep Disorders Centre, University of Manitoba, Winnipeg, Canada) and sleepFFT (Biosoft Studio, Pennsylvania State University, Hershey, PA). MSS analyzed central sleep spindles and sleepFFT central σ power.

### Sleep spindles

All sleep EEGs were analyzed with MSS through a data use agreement between J.F.M. (Pennsylvania State University) and M.Y. (University of Manitoba). De-identified EDF files were securely shared with M.Y. blind of any demographic, clinical or date of recording data to assure blindness and rigor of EEG data processing. MSS provided spindle metrics (density, frequency, power, and fast spindle percent) for N2 sleep in central derivations (C3 and C4), and were identified in the 10.0 to 16.0 Hz range. Details on this validated software can be found elsewhere (Guadagni et al., 2021; Goldschmied et al., 2020). In brief, the fast Fourier transform (FFT) was applied to 1-s epochs with the window advancing every 0.2 s (five windows per second). The sum of power in the spindle frequency range (power S) was calculated in each 1-s epoch. The spindle frequency range was defined as 10.0–16.0 Hz. Power S in each 1-s epoch was divided by the 30th percentile of power S in all 1-s epochs within each 30-s epoch (power S ratio). A spindle was identified when the power S ratio was >3 for five consecutive epochs provided the ratio decreased to <1.5, or power S decreased to <20% of peak power S (whichever was higher) within 5 s (Goldschmied et al., 2020). Presumptive spindles were deleted if they occurred during arousals and if the power S ratio within the presumptive spindle was less than the ratios of α and β powers to their respective reference values (30th percentile in the 30-s epoch). Spindle density was calculated as the total number of spindles in N2 sleep and divided by the time in minutes of N2 sleep. Spindle frequency was measured as the frequency with the highest power in the 10.0 to 16.0 Hz range and expressed in Hertz. Spindle power was defined as the highest power S within the spindle and expressed as microvolts squared (μV^2^). Fast spindles were identified in the 12.0 to 16.0 Hz range and expressed as the percent of fast spindles out of all identified spindles. Averages of spindle density, frequency, power, and fast spindle percent were calculated in N2 for central derivations.

### σ power

All sleep EEGs were also analyzed with sleepFFT software (Fernandez-Mendoza et al., 2016, 2019; Ricci et al., 2021) in which the FFT was used to estimate absolute NREM (N2 and N3) σ power. σ power was defined as frequencies in the 11.33 to 14.84 Hz range, consistent with previous studies using the frequency range of 11–15 Hz (Tarokh et al., 2011; Campbell and Feinberg, 2016; Purcell et al., 2017). σ power was analyzed in addition to spindle metrics to increase the rigor and reproducibility of the present study as it has been used in previous studies as a surrogate measure of spindle density and may follow a similar age-related trajectory. The same central EEG derivations (C3 and C4) were analyzed in all artifact-free NREM sleep epochs. All EDF files underwent thorough systematic procedures for rejecting EEG epochs with movement artifacts, correcting ECG interference intruding into EEG channels, sorting spectral data according to visually scored sleep stages, and calculation of EEG power during sleep/wake states using the FFT with correction for rejected epochs. We verified our automatic processing methods by visual inspection at epoch-by-epoch levels and compared automatic processing to visual inspection results in 1063 subjects (643 baseline and 420 follow-up) to assure the rigor and reproducibility of our data. Trained technicians examined the automatic processing results at an epoch-by-epoch level and had the ability to make corrections. The results of visual inspection by trained technicians were compared with automatic processing results in 1063 subjects, which resulted in high concordance (0.93–1.0), indicating that these methods highly agree with each other. We also determined the percent of epochs included in the analyses across all subjects, which was 85–90% across all sleep stages and channels. Therefore, automatic processing provided valid representations of our data that agree with human visual inspection.

All-night spectral analysis was performed on visually scored 30-s epochs. All overnight PSG data were automatically processed in a single run with sleepFFT by a trained graduate assistant blind of any of the subject’s characteristics. As mentioned above, the common EEG frequencies ranged from 0.3–30.0 Hz and activities below and above this range were removed for consistent data processing across all EDF records from different PSG systems (B1, B2, and F). SleepFFT used eight orders of Butterworth band pass filter with a high pass filter set at 0.3 Hz and a low pass filter set at 30.0 Hz. Each 30-s epoch was applied with 22 overlapping Hann windows lasting 2.56 s, with overlaps between windows by approximately half. The FFT was performed on each overlapping window to generate power density data with 0.39 Hz resolution. The resulting data were averaged across these 22 windows as the power spectral data for the epoch. Absolute NREM (N2 and N3) σ power was computed by summing the power density data (including lower and upper limits of the frequency band), adjusting for rejected epochs, averaging for C3 and C4, and expressed as μV^2^.

### Statistical analyses

In order to examine the age-related trajectories of spindle/σ activity we derived a cross-sectional sample spanning from age 5 to 23 by aggregating independent subjects who contributed with data at ages 5–12 years (*n* = 279) and at ages 12–23 years (*n* = 421) with no subject represented twice. After excluding participants who were recorded on paper PSG (*n* = 27), whose EDF file was corrupted (*n* = 3), who had missing spectral data or were outliers (*n* = 6), or were taking psychotropic medications (*n* = 92), 572 subjects were included in the cross-sectional analyses. Multivariable-adjusted linear regression models regressed subjects’ age against spindle/σ activity. Age was treated as a continuous variable and truncated at 6 and 21 years because only two subjects were 5 years and eight were ≥22 years. Given that previous studies (Nader and Smith, 2015; McClain et al., 2016; Purcell et al., 2017) found different, nonlinear age trajectories for specific spindle metrics, we tested nonlinear associations between age and spindle/σ activity by including quadratic and cubic terms in the models, along with the lower-order terms. The highest-ordered significant (*p* < 0.05) age term was used as the final model. The population-level means and their 95%CI of spindle/σ activity between ages 6 and 21, estimated based on the final model, were plotted to represent the cross-sectional age-related trajectories. Covariates adjusted for in these models included sex, race/ethnicity, BMI, apnea/hypopnea index (AHI), psychiatric/learning disorder and PSG system (coded as B1 = 0, B2 = 1, F = 2 and treated as a nominal factor). Sex-specific distributions were plotted for the highest significant ordered term. Furthermore, we estimated the age at which minimum and maximum predictive values in spindle/σ activity were reached. Piece-wise linear regression analyses were performed to obtain standardized regression coefficients (β_s_) in the association between age and spindle/σ activity. Spindle/σ activities were divided by their own standard deviation to obtain the β_s_ and their standard error (SE). Sex differences in mean spindle/σ activity were also tested using analysis of covariance at developmentally appropriate piece-wise age segments [6–10 (childhood, *n* = 188), 11–14 (early adolescence, *n* = 108), 15–17 (mid-adolescence, *n* = 162), and 18–21 (late adolescence/early adulthood, *n* = 114)].

In order to study the magnitude of longitudinal change in spindle/σ activity in the transition from childhood to adolescence, we focused on a sample of 332 subjects who had analyzable sleep EEG data at ages 5–12 years (baseline) and at ages 12–22 years (follow-up) and were not taking psychotropic medications. The within-subject change in spindle/σ activity between baseline and follow-up was the dependent variable in these longitudinal analyses and was calculated as a percent change with the formula: [(follow-up value – baseline value)/baseline value] × 100. General linear models were used to calculate the age-related percent change in spindle/σ activity as a function of the following age groups: 12–14 years (early adolescence, *n* = 75), 15–17 years (mid-adolescence, *n* = 160), and 18–22 years (late adolescence/early adulthood, *n* = 97). Covariates adjusted for in these longitudinal models included sex, race/ethnicity, BMI, AHI, psychiatric/learning disorders, baseline PSG system (coded as B1 = 0, B2 = 1 and treated as a binary factor), baseline spindle/σ activity, and length of follow-up (years elapsed between baseline and follow-up). By examining the longitudinal trajectory using this approach, we calculated the change in spindle/σ activity in the transitions from baseline mean age of 6.6 years to follow-up age 12–14 years, from 8.7 to 15–17 years and from 10.3 to 18–22 years, which allows for comparison with previous experimental studies estimating the longitudinal change within similar transitions (Tarokh and Carskadon, 2010; Tarokh et al., 2011; Hahn et al., 2019). Sex differences in mean spindle/σ activity were tested using analysis of covariance at each age group. In addition, pubertal-related differences in mean spindle/σ activity between subjects reporting Tanner stages 1–3 (*n* = 56) versus 4–5 (*n* = 256) were tested at each age group, except at ages 18–22 as only one subject ≥18 years reported a Tanner stage 3 and was removed from this analysis as an outlier. The results of these models are expressed as multivariable-adjusted means (95%CI). Statistical analyses were performed using SAS version 9.4 (SAS Institute).

## Results

### Cross-sectional trajectories

The cross-sectional sample consisted of 572 subjects aged 6–21 (truncated), of whom 48% were female and 26% were a racial/ethnic minority (Table 1). Mean PSG parameters were commensurate with the age range of the sample whose total sleep time (TST) was 449 min, of which 51% was spent in N2 and 81% in NREM sleep.

**Table 1:** Characteristics of the cross-sectional and longitudinal samples

	Cross-sectional^*a*^	Longitudinal (*N* = 332)^*b*^	
	(*N* = 572)	Baseline	Follow-up
Sex			
Male	300 (52.4%)	178 (53.6%)	178 (53.6%)
Female	272 (47.6%)	154 (46.4%)	154 (46.4%)
Race/ethnicity			
Non-Hispanic White	424 (74.1%)	252 (75.9%)	252 (75.9%)
Racial/ethnic minority	148 (25.9%)	80 (24.1%)	80 (24.1%)
Age			
5	2 (0.3%)	6 (1.8%)	
6	32 (5.6%)	35 (10.5%)	
7	38 (6.6%)	53 (16.0%)	
8	48 (8.4%)	51 (15.4%)	
9	32 (5.6%)	72 (21.7%)	
10	36 (6.3%)	57 (17.2%)	
11	28 (4.9%)	51 (15.4%)	
12	13 (2.3%)	7 (2.1%)	8 (2.4%)
13	27 (4.7%)		27 (8.1%)
14	40 (7.0%)		40 (12.0%)
15	50 (8.7%)		51 (15.4%)
16	59 (10.3%)		58 (17.5%)
17	53 (9.3%)		51 (15.4%)
18	46 (8.0%)		45 (13.6%)
19	31 (5.4%)		27 (8.1%)
20	21 (3.7%)		15 (4.5%)
21	8 (1.4%)		7 (2.1%)
22 23	7 (1.2%) 1 (0.2%)		3 (0.9%)
BMI percentile			
Normal weight	373 (65.2%)	232 (69.9%)	225 (67.8%)
Overweight	113 (19.8%)	36 (10.8%)	61 (18.4%)
Obese	86 (15.0%)	64 (19.3%)	46 (13.9%)
Behavioral disorders			
None	449 (78.5%)	274 (82.5%)	248 (74.7%)
Yes	123 (21.5%)	58 (17.5%)	84 (25.3%)
Neurobehavioral measures			
Attention problems, score	54.6 (6.6)	54.3 (6.4)	54.8 (6.6)
Thought problems, score	55.1 (6.6)	55.4 (6.9)	54.5 (5.8)
Internalizing problems, score	50.5 (10.7)	50.6 (10.7)	50.4 (10.0)
Externalizing problems, score	48.1 (10.8)	48.3 (11.0)	48.2 (10.0)
Verbal IQ, score	105.8 (13.2)	107.0 (13.2)	102.0 (10.4)
Non-verbal IQ, score	106.4 (15.5)	108.1 (15.5)	106.1 (11.6)
Coding, scaled score	10.3 (3.2)	10.6 (3.3)	9.8 (2.6)
Digit span backward, raw score	4.5 (1.7)	4.6 (1.6)	6.9 (2.4)
PSG			
SOL, min	27.4 (22.2)	26.8 (22.3)	25.0 (20.0)
WASO, min	61.6 (41.0)	43.9 (32.9)	69.2 (41.1)
TST, min	448.6 (51.3)	461.3 (44.3)	448.0 (49.8)
SE, %	83.6 (9.3)	86.7 (7.6)	82.9 (9.1)
N1, %	2.0 (2.7)	3.5 (2.6)	0.9 (1.1)
N2, %	51.1 (10.9)	46.7 (10.8)	53.2 (9.4)
N3, %	27.9 (10.1)	29.9 (10.5)	27.1 (9.0)
R, %	19.0 (5.2)	20.1 (5.3)	18.8 (4.7)
AHI, events/h	1.9 (4.7)	0.8 (1.1)	2.5 (5.7)

a Data for 572 subjects in cross-sectional analyses.

b Data for 332 subjects in longitudinal analyses, including baseline and follow-up.

Data are means (SD) and number of cases (percentage) for continuous and categorical/ordinal variables, respectively. AHI = apnea/hypopnea index; BMI = body mass index; N1 = epochs scored as NREM stage 1; N2 = epochs scored as NREM stage 2; N3 = epochs scored as NREM stages 3 or 4; R = epochs scored as REM sleep; SE = sleep efficiency; SOL = sleep onset latency; TST = total sleep time; WASO = wake after sleep onset.

**Figure 1.**
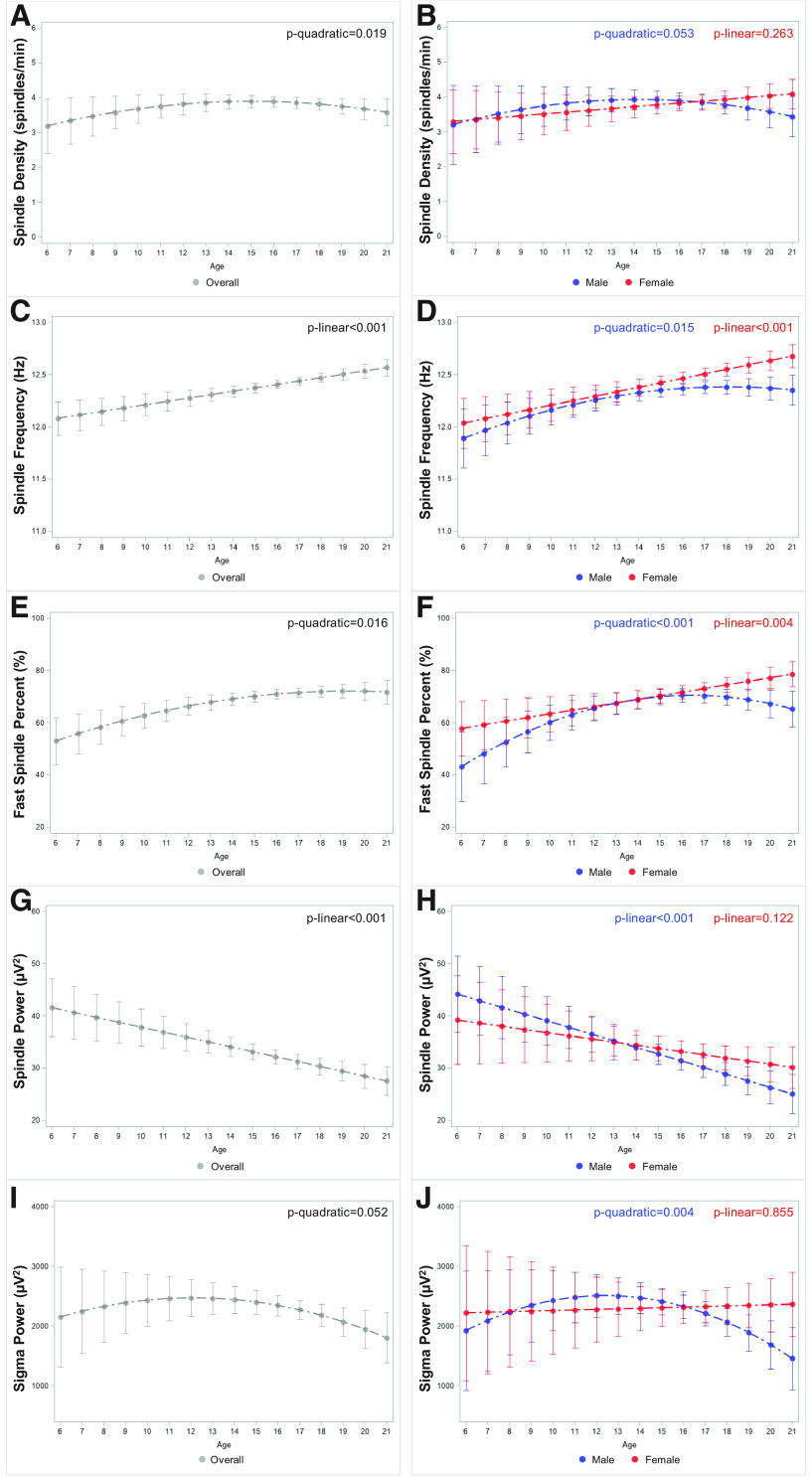
Cross-sectional trajectories of sleep spindles and sigma power in 572 subjects aged 6–21. Data points are multivariable-adjusted means and their 95% CI and lines are multivariable-adjusted regression curves for sleep spindle and sigma activity. ***A***, Spindle density (# of spindles/minute) overall. ***B***, Spindle density stratified by sex. ***C***, Spindle frequency (Hz) overall. ***D***, Spindle frequency stratified by sex. ***E***, Fast spindle percent (% of spindles in the 12–16 Hz range) overall. ***F***, Fast spindle percent stratified by sex. ***G***, Spindle power (μV2) overall. ***H***, Spindle power stratified by sex. ***I***, Sigma power (μV2) overall. ***J***, Sigma power stratified by sex.

As shown in Figure 1*A*, the age-related trajectory of spindle density was best fit by a quadratic model (*R*^2^= 0.106). Spindle density was lowest at age 6.0 and highest at age 15.2; specifically, spindle density increased between ages 6 and 14 [β_s_ (SE)= 0.124 (0.051), *p* = 0.014] and remained stable between ages 15 and 21 [β_s_ (SE)= 0.009 (0.049), *p* = 0.852]. The quadratic trajectory of spindle density was significant for males (*R*^2^= 0.107), while it did not reach statistical significance in cubic, quadratic, nor linear (*R*^2^= 0.105) models for females (Fig. 1*B*). Spindle density was lowest at age 6.0 and highest at age 14.2 in males. There were no statistically significant differences in spindle density between males and females when examining piece-wise age segments 6–10 years (*p* = 0.100), 11–14 years (*p* = 0.878), 15–17 years (*p* = 0.688), or 18–21 years (*p* = 0.223).

As observed in Figure 1*C*, the age-related trajectory of spindle frequency was best fit by a linear model (*R*^2^= 0.212), by which it increased from age 6.0 (lowest) to 21.0 (highest). While this linear trajectory was significant in females (*R*^2^= 0.246), males experienced a quadratic trajectory (*R*^2^= 0.191) in spindle frequency (Fig. 1*D*). Males reached a peak in spindle frequency at age 18.0 and females at age 21.0. Commensurate, females experienced a steeper increasing slope in spindle frequency than males between ages 18 and 21 [β_s_ (SE)= 0.155 (0.062) in females vs −0.031 (0.058) in males, p for interaction = 0.013]. Spindle frequency was higher in females at ages 6–10 (*p* = 0.015) and at ages 18–21 (*p* = 0.006) but not at ages 11–14 (*p* = 0.733) or 15–17 (*p* = 0.347).

The age-related trajectory for the percent of fast spindles was best fit by a quadratic model (*R*^2^= 0.177; Fig. 1*E*). Fast spindle percent was lowest at age 6.0 and highest at age 19.3 [β_s_ (SE) between ages 6 and 19 = 1.562 (0.450), *p* < 0.001]. This quadratic trajectory was significant in males (*R*^2^= 0.173), while fast spindle percent followed a linear (*R*^2^= 0.189) age-related trajectory in females (Fig. 1*F*). Although the lowest percent of fast spindles occurred at age 6.0 in males and females, males reached their peak in fast spindle percent at age 16.5 and females at age 21.0. Females showed a higher percent of fast spindles than males at ages 6–10 (*p* = 0.030) and 18–21 (*p* = 0.007) but not at ages 11–14 (*p* = 0.693) or 15–17 (*p* = 0.809).

As observed in Figure 1*G*, the age-related trajectory of spindle power was best fit by a linear model (*R*^2^= 0.516), as it decreased from age 6.0 (highest) to 21.0 (lowest). However, males showed a steeper decreasing slope than females from age 6–21 [β_s_ (SE)= −1.244 (0.314), *p* linear < 0.001; *R*^2^= 0.514 in males vs β_s_ (SE)= −0.431 (0.391), *p* linear = 0.271; *R*^2^= 0.520 in females;Fig. 1*H*]. Spindle power was higher in females at ages 6–10 (*p* = 0.045) and 18–21 (*p* < 0.001) but not at ages 11–14 (*p* = 0.462) or 15–17 (*p* = 0.136).

Similar to spindle density and fast spindle percent, we found a significant quadratic trajectory (*R*^2^= 0.235) for σ power (Fig. 1*I*). However, highest σ power was observed at age 13.0 and lowest at age 21.0; specifically, σ power decreased between ages 13 and 21 [β_s_ (SE)= 73.235 (25.773), *p* = 0.005]. Similar sex-related trajectories to those observed in spindle density were found for σ power, where a quadratic trajectory (*R*^2^= 0.226) was significant for males, while it did not reach statistical significance in cubic, quadratic nor linear (*R*^2^= 0.246) models for females (Fig. 1*J*). In males, σ power was highest at age 12.6 and lowest at age 21.0. σ power was greater in females than males at ages 6–10 (*p* = 0.033) and 18–21 (*p* = 0.014) but not ages 11–14 (*p* = 0.491) or 15–17 (*p* = 0.611).

Table 2 shows that, while σ power was weakly correlated with spindle density (*r* = 0.311), it was moderately correlated with spindle power (*r* = 0.628). Spindle density and spindle power were moderately correlated to each other (*r* = 0.553), while spindle frequency was strongly correlated with fast spindle percent (*r* = 0.931).

**Table 2:** Correlation coefficients for sleep spindle/σ activity in the cross-sectional sample (above diagonal) and the longitudinal sample (below diagonal)

	Spindle density	Spindle frequency	Fast spindle percent	Spindle power	σ power
Spindle density	---	0.205	0.263	0.553	0.311
Spindle frequency	0.286	---	0.931	−0.120	−0.104
Fast spindle percent	0.349	0.564	---	−0.061	−0.106
Spindle power	0.471	0.191	0.248	---	0.628
σ power	0.553	0.262	0.225	0.671	---

Data are Pearson correlation coefficients. For the cross-sectional sample, the correlation coefficients for spindle density (#/min), spindle frequency (Hz), fast spindle percent (%), and spindle and σ power (μV^2^) were determined. For the longitudinal sample, the correlation coefficients for the percent change in each spindle metric from baseline (ages 5–12) to follow-up (ages 12–22) were calculated.

### Longitudinal trajectories

The longitudinal sample consisted of 332 children aged 5–12 at baseline (46% female, 24% racial/ethnic minority) who were followed-up 6–13 years later at ages 12–22 (Table 1). PSG parameters changed in the expected direction from baseline to follow-up, with TST decreasing and percent N2 increasing.

**Figure 2.**
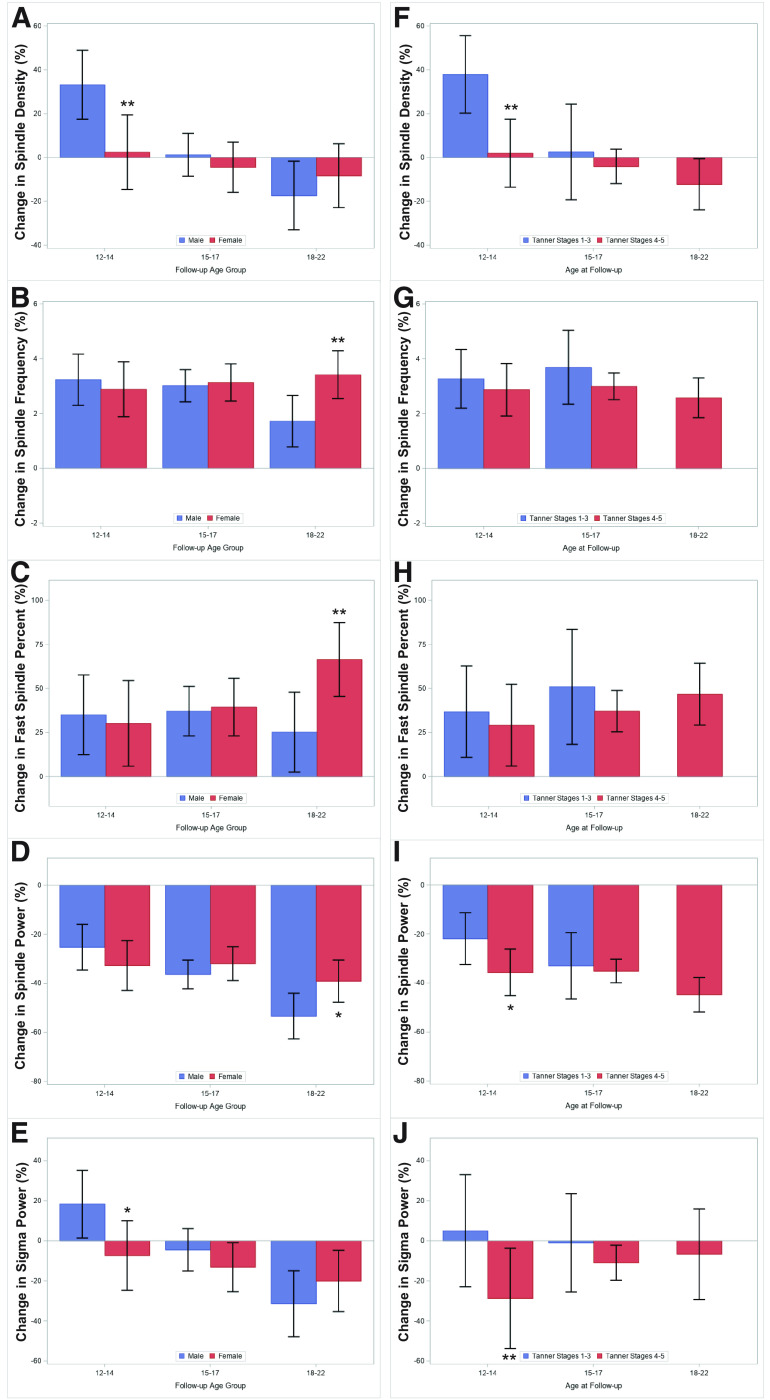
Sex (left column) and Tanner-related (right column) differences in the longitudinal trajectories of sleep spindles and sigma power in 332 subjects aged 5–12 at baseline followed-up at ages 12–22. Data are multivariable-adjusted percent change (95% CI) from baseline to follow-up in sleep spindle and sigma activity. Age groups represent transitions between baseline age (mean) 6.6 years to follow-up ages 12–14, from 8.7 years to ages 15–17, and from 10.3 years to ages 18–22. ***A***, Spindle density stratified by sex. ***B***, Spindle frequency stratified by sex. ***C***, Fast spindle percent stratified by sex. ***D***, Spindle power stratified by sex. ***E***, σ power stratified by sex. ***F***, Spindle density stratified by Tanner stage. ***G***, Spindle frequency stratified by Tanner stage. ***H***, Fast spindle percent stratified by Tanner stage. ***I***, Spindle power stratified by Tanner stage. ***J***, σ power stratified by Tanner stage. *p < 0.05, **p < 0.01.

There was a significant age effect in the longitudinal change of spindle density from baseline to follow-up (*p* < 0.001; *R*^2^= 0.270). As seen in Figure 2*A*, males had experienced a greater longitudinal increase in spindle density (33.2%, 95%CI = 17.4%, 48.9%) than females (2.4%, 95%CI = −14.7%, 19.5%) when followed-up at ages 12–14 (*p* = 0.006). There were no statistically significant sex differences in spindle density in the transition to ages 15–17 (*p* = 0.458) or 18–22 (*p* = 0.364).

There was an overall longitudinal increase in spindle frequency (Fig. 2*B*) and fast spindle percent (Fig. 2*C*) with no statistically significant age effect (*p* = 0.087, *R*^2^= 0.163 and *p* = 0.665, *R*^2^= 0.368, respectively). Females had experienced a greater longitudinal increase in spindle frequency (3.4%, 95%CI = 2.5%, 4.3%) than males (1.7%, 95%CI = 0.8%, 2.7%) when followed-up at ages 18–22 (*p* = 0.004; Fig. 2*B*). Further, females had experienced a greater increase in fast spindle percent (66.4%, 95%CI = 45.4%, 87.4%) than males (25.2%, 95%CI = 2.6%, 47.9%) when followed-up at ages 18–22 (*p* = 0.004; Fig. 2*C*). There were no statistically significant sex differences in spindle frequency nor fast spindle percent in the transitions to ages 12–14 (*p* = 0.598 and *p* = 0.759, respectively) nor 15–17 (*p* = 0.795 and *p* = 0.833, respectively).

There was an overall longitudinal decline in spindle power with a significant age effect (*p* < 0.001; *R*^2^= 0.330). Males had experienced a greater decline in spindle power (−53.4%, 95%CI= −62.7%, −44.0%) than females (−39.1%, 95%CI= −47.7%, −30.5%) when followed-up at ages 18–22 (*p* = 0.018; Fig. 2*D*). There were no statistically significant sex differences in spindle power in the transitions to ages 12–14 (*p* = 0.265) nor 15–17 (*p* = 0.341).

There was also a significant age effect (*p* < 0.001; *R*^2^= 0.264) in the longitudinal change in σ power from baseline to follow-up. As shown in Figure 2*E*, males had experienced a 26% greater increase in σ power (18.3%, 95%CI = 1.4%, 35.2%) than females (−7.4%, 95%CI= −24.7%, 10.0%) when followed-up at ages 12–14 (*p* = 0.032). There were no statistically significant sex differences in σ power in the transitions to ages 15–17 (*p* = 0.291) nor 18–22 (*p* = 0.280).

We also examined the longitudinal change in spindle and σ activity as a function of Tanner stage at follow-up. As observed in Figure 2*F*, subjects who reported Tanner stages 1–3 had experienced a greater longitudinal increase in spindle density (37.9%, 95%CI = 20.2%, 55.6%) than those reporting Tanner stages 4–5 (1.9%, 95%CI= −13.5%, 17.4%) when followed-up at ages 12–14 (*p* = 0.002) but not at ages 15–17 (*p* = 0.575). As seen in Figure 2*G*,*H*, there were no significant differences between subjects reporting Tanner stages 1–3 versus 4–5 at ages 12–14 or 15–17 in the longitudinal change of spindle frequency (*p* = 0.576 and *p* = 0.340, respectively) or fast spindle percent (*p* = 0.661 and *p* = 0.432, respectively). Subjects who reported Tanner stages 4–5 had experienced a 14% greater longitudinal decline in spindle power (−35.7%, 95%CI= −45.2%, −26.1%) than those reporting Tanner stages 1–3 (−21.9%, 95%CI= −32.5%, −11.3%) when followed-up at ages 12–14 (*p* = 0.055) but not at ages 15–17 (*p* = 0.769; Fig. 2*I*). Lastly, subjects who reported Tanner stages 4–5 had experienced a 34% greater longitudinal decline in σ power (−28.8%, 95%CI= −53.8%, −3.7%) than those reporting Tanner stages 1–3 (5.0%, 95%CI= −23.0%, 33.0%) when followed-up at ages 12–14 (*p* = 0.008) but not at ages 15–17 (*p* = 0.448; Fig. 2*J*).

Finally, as shown in Table 2, the longitudinal change in σ power was moderately correlated with both the change in spindle density (*r* = 0.553) and in spindle power (*r* = 0.671) from baseline to follow-up. The change in spindle density was weakly correlated with the change in spindle power (*r* = 0.471), while the changes in spindle frequency and fast spindle percent were moderately correlated (*r *= 0.564) to each other.

## Discussion

This study delineates sex-related and pubertal-related differences in the maturational trajectories of sleep spindles in a population-based cohort capturing the critical developmental period between childhood and adolescence. We provide robust evidence that sleep spindle metrics follow distinct developmental trajectories that deviate from other sleep EEG oscillations previously associated with brain maturation, namely slow wave activity (SWA) in the δ-frequency (0.4–4.0 Hz) range (Baker et al., 2012; Campbell et al., 2012; Campbell and Feinberg, 2016; Gorgoni et al., 2020; Ricci et al., 2021). Specifically, spindle density, fast spindle percent, and σ power, follow inverted U-shaped trajectories from age 6 to 21, by which both spindle density and σ power increase during childhood, peak in mid-adolescence, and begin to decline in late adolescence/early adulthood. While the percent of fast spindles also increases in childhood, it continues to increase further into late adolescence, commensurate with the linear increase in spindle frequency from age 6 to 21. In contrast, spindle power linearly declines from childhood to early adulthood. Overall, our novel data show that males experience greater maturational changes in spindle density, spindle power and σ power, which is driven by their later pubertal development, yet sex differences become prominent in early adulthood when males have lower spindle frequency, spindle power and σ power as well as a lower percentage of fast spindles.

Our observed inverted U-shaped cross-sectional trajectory of spindle density from childhood to early adulthood replicates previous life-course analyses by Purcell et al. (2017), by which spindle density increases in childhood, peaks in adolescence, and starts to decline in adulthood. Although both σ power and spindle density increase during childhood and peak in adolescence, the peak in σ power occurs two years earlier (age 13) than spindle density (age 15). In addition, σ power declines to a greater extent in early adulthood and does not reach its lowest until age 21, while lowest spindle density occurs at age 6. Further, the decline in σ power in late adolescence/early adulthood is consistent with and may be related to the decline in spindle power observed through age 21 as they are moderately correlated. Thus, although σ power may undergo similar age-related changes to spindle density and spindle power, using σ power as a surrogate marker for all spindle metrics may mask specific age-related changes in important spindle metrics, including density, as shown by the small to moderate correlations in Table 2 between σ power and spindle activity (Goldstone et al., 2019; Gorgoni et al., 2020).

From a neurobiological standpoint, the observed increase in spindle density up to mid-adolescence may represent the increased myelination of thalamocortical projections that occurs with typical neurodevelopment (Steriade et al., 1985; Tarokh et al., 2011), as neuroimaging studies have shown higher spindle density to be associated with enhanced white matter diffusion along axons (Piantoni et al., 2013). Additionally, Hahn et al. (2019) suggested that the increase in spindle density in adolescence may be related to the emergence of centroparietal fast spindles. Consistently, the inverted-U shaped trajectory in spindle density coupled with the linear increase in spindle frequency was reflected in the percent of fast spindles also increasing in early and mid-adolescence and peaking by age 19, suggesting that “full” maturation of the thalamocortical network may occur by late adolescence, consistent with previous neuroimaging studies examining maturational changes in cortical gray and white matter (Giedd et al., 1999; Giorgio et al., 2010). Furthermore, the increase in spindle frequency from childhood to early adulthood is hypothesized to reflect reduced hyperpolarization of thalamocortical neurons that occurs with the decline in sleep depth throughout adolescence (Campbell and Feinberg, 2016; Goldstone et al., 2019). This reduced hyperpolarization of thalamocortical projections increases the number of neurons able to produce spindles, thus, allowing for greater spindle density and spindle frequency (Steriade et al., 1985; Goldstone et al., 2019). Further, Saletin et al. (2013) found fast (13–15 Hz) spindle frequencies to be positively associated with hippocampal gray matter volume, which was a predictor of increasingly fast frequency spindles in 22 young adults. Fast spindles have also been associated with hippocampal-dependent memory consolidation (Diekelmann and Born, 2010), thus, the increase in spindle frequency and percent of fast spindles throughout childhood and adolescence may also reflect maturation of hippocampal connectivity (Saletin et al., 2013; Astill et al., 2014). Our observed age-related trajectories of spindle density, frequency, and percent of fast spindles, taken together with previous EEG and neuroimaging studies, further support the role of sleep spindles as EEG biomarkers of thalamocortical and hippocampal development.

An important novel aspect of our study was examining within-subjects changes in spindle metrics as a function of pubertal development, as previous studies have been limited by short follow-up periods in childhood and adolescence and a lack of pubertal data (Purcell et al., 2017; Hahn et al., 2019). Adolescents reporting more mature pubertal stages experienced a greater decline in σ and spindle power than less mature adolescents in the transition from childhood (ages 5–8, mean 6.6 years) to early adolescence (ages 12–14, mean 13.4 years). These data suggest that changes in σ and spindle power may be closely related to pubertal development similar to SWA power, a well-described marker of brain maturation, specifically synaptic pruning (Baker et al., 2012; Feinberg and Campbell, 2012; Ricci et al., 2021). This pubertal-related decline in σ and spindle power observed in more mature youth in the transition to early adolescence suggests a potential role for synaptic pruning, which may be impacting the synchronization of slow wave and sleep spindle oscillations at the cortical level because of their similar origin in the thalamocortical network (Steriade, 2006; Campbell and Feinberg, 2016; Gorgoni et al., 2020). Furthermore, spindle and σ power have been associated with subcortical white matter integrity around the thalamus in adults (Piantoni et al., 2013; Gaudreault et al., 2018; Fernandez and Lüthi, 2020); thus, an additional neurobiological mechanism that may be driving the decline in spindle and σ power in adolescence may be reduced diffusivity around the neuronal membrane, which leads to greater neural synchrony in thalamocortical loops (Gaudreault et al., 2018). Although both spindle and σ power decline in the transition to adolescence, spindle power continues to decline further into late adolescence/early adulthood in mature subjects (−45% decline in spindle power by 18–22 years vs −7% in σ power). Taken together, these data suggest that the decrease in σ power may be more related to cortical synaptic pruning in the transition to adolescence, while the decline in spindle power may reflect the continued maturation of subcortical white matter (Campbell and Feinberg, 2016; Gaudreault et al., 2018). We also found that adolescents reporting less mature pubertal stages, 86% of whom were males, had experienced a greater increase in spindle density in the transition to early adolescence (age 12–14), while more mature adolescents, 75% of whom were females, had already experienced such increase in spindle density. Overall, these data suggest that females, who enter pubertal development earlier than males, may also begin brain maturational processes in the thalamocortical network at an earlier stage than males (Giedd et al., 1999; Colrain and Baker, 2011). Interestingly, there were no significant pubertal-related differences in spindle frequency nor percent of fast spindles, suggesting that while the quantity (density) of spindles generated by the thalamus is associated with pubertal development, the quality (frequency) of spindles expressed at the cortical level may not (Anderer et al., 2001; Saletin et al., 2013). Future studies examining age-related changes in cortical and subcortical gray and white matter and sleep spindle/σ activity are needed to shed further light on the association of spindle/σ activity with specific brain maturational processes.

As it pertains to sex differences, males appeared to drive the inverted U-shaped cross-sectional trajectories of spindle density, fast spindle percent, and σ power. Females maintained more stable levels of spindle density and σ power from childhood into early adulthood, while males experienced greater maturational changes. Although males reached peak σ power and spindle density at ages 12.6 and 14.2, they experienced steeper declines throughout late adolescence/early adulthood leading to lower σ power than females, consistent with Purcell et al. (2017). The longitudinal change in spindle density by ages 12–14 was lower in females than males, suggesting that females had already experienced their maximum spindle density before the transition to early adolescence, commensurate with our observed pubertal differences. Similarly, males appeared to drive the linear decline in spindle power in late adolescence/early adulthood, as they exhibited a steeper maturational slope and greater longitudinal decline than females by ages 18–22. Together, these findings indicate that the age-related trajectories of spindle density, spindle power and σ power are associated with the onset of puberty, which occurs earlier in females than males (Giedd et al., 1999; Colrain and Baker, 2011). Thus, females may mature their spindle characteristics at earlier developmental stages, while males experience greater maturational changes through early adulthood, consistent with a previous study by Ujma et al. (2016), suggesting that spindle density is a maturational marker in males, but may be a trait-like EEG feature in females. Interestingly, females experienced a linear increase in spindle frequency, leading them to have a higher percentage of fast spindles than males by early adulthood, commensurate with previous studies in adolescents and adults (Purcell et al., 2017; Goldstone et al., 2019). While the percent of fast spindles plateaued in males by age 17, it continued to increase in females through age 21. These data suggest that females may have greater thalamocortical connectivity and coherence than males by early adulthood (Markovic et al., 2020). Overall, the observed sex differences in spindle activity indicate that there may be potential differences in thalamocortical loops in females and males (Markovic et al., 2020); however, future studies that examine brain connectivity, cognitive processes, and sleep spindles are needed to shed further light on the functional significance of the observed maturational sex differences.

Several potential limitations of the present study must be discussed. Sleep studies consisted of a one-night PSG recording, which may be affected by the first night effect; however, spectral EEG measures have shown greater night-to-night stability than traditional sleep continuity/architecture parameters, assuring the generalizability of the findings (Tan et al., 2000; Israel et al., 2012). Although we were able to control for AHI, immediate sleep history during the week previous to the in-lab study was not available and standardized across all age groups, and thus, could not be controlled for in the analyses. Additionally, there were PSG system updates during this long-term study; however, we accounted for each setting during EEG data processing and controlled for the PSG system used in statistical analyses to mitigate the potential impact on the estimation and trajectories of sleep spindles and σ activity. The lack of a significant age term (linear, quadratic, or cubic) for spindle density, spindle power or σ power in females, may be because of a lack of statistical power. Although some subjects contributed with ipsilateral central derivations at ages 5–12 and and the distance between active and reference electrode can affect the amplitude of spectral bands, there was excellent concordance between contralateral and ipsilateral derivations, assuring the validity of our data (see Materials and Methods, PSG). Our longitudinal analyses in 332 subjects were based on two time-points, which precluded analyzing the data with longitudinal growth curves. Although we had Tanner staging available at follow-up, it was not collected at baseline; however, the age-sex-Tanner distribution was that expected for a population-based sample where females mature earlier than males (Campbell et al., 2012), assuring the generalizability of our findings. Additionally, we focused our analyses on central derivations as frontal derivations (e.g., F3-M2) were not available at both time points, which precluded topographical analysis of spindle metrics in the cross-sectional and longitudinal trajectories for either the entirety of the 6–21 life course or in the transition from ages 5–12 to ages 12–22. However, spindle activity has been shown to be best quantified at central derivations (Goldschmied et al., 2020), assuring the reliability of our findings. Finally, although we were able to calculate the percent of fast spindles, MSS software did not allow estimating the density, power, or peak frequency of fast and slow spindles separately, thus, future studies are necessary to determine the age-related trajectories of those specific characteristics.

In conclusion, we provide population-based evidence that sleep spindle metrics follow distinct developmental trajectories from each other and from other synchronized oscillations of NREM sleep previously shown to index brain maturation (e.g., SWA power) during the critical transition from childhood to adolescence. Although sleep spindles occur within the σ-frequency range, it appears that using σ power as a surrogate marker for spindle activity may mask spindle-specific maturational changes. The increase in spindle density in childhood and peak in mid-adolescence coupled with the linear increase in spindle frequency may represent the increasing predominance of fast spindles and, thus, maturation of thalamocortical and hippocampal connectivity, which appears to continue until late adolescence. The greater longitudinal decline in σ and spindle power in more mature adolescents as well as the greater increase in spindle density in less mature subjects by early adolescence indicates that females, who enter pubertal development earlier than males, may begin maturation of their thalamocortical loops earlier than males. Indeed, females had higher spindle frequency, spindle power and σ power and a greater percentage of fast spindles by early adulthood, which may reflect greater thalamocortical coherence, while males experience greater maturational changes in spindle density, spindle power and σ power throughout childhood and adolescence. These data have important implications for future studies examining the role of sleep spindle activity in specific psychiatric and/or learning disorders as well as their relationship to cognitive abilities in youth at different developmental stages.

## Synthesis

Reviewing Editor: Arianna Maffei, SUNY Stony Brook Decisions are customarily a result of the Reviewing Editor and the peer reviewers coming together and discussing their recommendations until a consensus is reached. When revisions are invited, a fact-based synthesis statement explaining their decision and outlining what is needed to prepare a revision will be listed below. The following reviewer(s) agreed to reveal their identity: NONE. Note: If this manuscript was transferred from JNeurosci and a decision was made to accept the manuscript without peer review, a brief statement to this effect will instead be what is listed below.

The authors thoroughly addressed the reviewers' comments with substantial editing and data analysis.

As the resolution of the figures is fine for evaluating the manuscript, but it is too low for publication, it would be helpful to provide figures with a higher resolution.
